# Essential Oils from *Papaver rhoeas* and Their Metabolomic Profiling

**DOI:** 10.3390/metabo14120664

**Published:** 2024-12-01

**Authors:** Valeria Cavalloro, Francesco Saverio Robustelli della Cuna, Alberto Malovini, Carla Villa, Cristina Sottani, Matteo Balestra, Francesco Bracco, Emanuela Martino, Simona Collina

**Affiliations:** 1 Department of Earth and Environmental Sciences, University of Pavia, Via Ferrata 1, 27100 Pavia, Italy; valeria.cavalloro@unipv.it (V.C.); francesco.bracco@unipv.it (F.B.); 2 National Biodiversity Future Center, Piazza Marina 61, 90133 Palermo, Italy; 3 Environmental Research Center, Istituti Clinici Scientifici Maugeri IRCCS, 27100 Pavia, Italy; francescosaverio.robustellidellacuna@unipv.it (F.S.R.d.C.); cristina.sottani@icsmaugeri.it (C.S.); 4 Laboratory of Informatics and Systems Engineering for Clinical Research, Istituti Clinici Scientifici Maugeri IRCCS, 27100 Pavia, Italy; alberto.malovini@icsmaugeri.it; 5 Department of Pharmacy, University of Genova, Viale Benedetto XV, 3, 16148 Genova, Italy; villa@difar.unige.it; 6 Department of Drug Sciences, University of Pavia, Via Taramelli 12, 27100 Pavia, Italy; matteo.balestra02@universitadipavia.it (M.B.); simona.collina@unipv.it (S.C.)

**Keywords:** *Papaver rhoeas*, solvent-free microwave extraction, essential oil, cantharidin, pollinator

## Abstract

**Background/Objectives**: Essential oils (EOs) have been exploited by humans for centuries, but many sources remain poorly investigated, mainly due to the low yields associated with conventional extraction. Recently, new techniques have been developed, like solvent-free microwave extraction (SFME), able to enhance efficiency and sustainability. The use of *Papaver rhoeas* L. in traditional medicine has led researchers to investigate non-volatile fractions, but there are little data available on EOs. **Methods**: In the present work, we prepared EOs from the petals and leaves of *P. rhoeas* by SFME. GC/MS analysis of EOs revealed the presence of 106 compounds belonging to 13 different classes. Isomers of the different alkenes were identified thanks to an alkylthiolation reaction. **Results**: The results highlighted a predominance of saturated and unsaturated hydrocarbons, alcohols, and esters that might suggest a specific relationship with pollinators. Each population has been compared using PCA, heatmap, and barplot tools, highlighting differences in terms of composition by both comparing leaves and flowers and hill and lowland samples. Furthermore, cantharidin, a metabolite usually produced by insects, was detected in the flowers, possible present for attractiveness purposes. **Conclusions**: These results could contribute to ensuring a better understanding of the pollination process and of the biological activities of EOs from *P. rhoeas*.

## 1. Introduction

Essential oils (EOs) are liquid hydrophobic mixtures containing volatile metabolites obtained from natural sources. The term “essential” is related to the fact that it contains the essence of the fragrance of the plant. The main functions of EOs are related to the interactions between the producing organisms and the environment, both considering plants and animals [1]. Thus, EOs provide scent and flavors and have a pivotal role in the relationship with pollinators and pests [2].

Due to the wide activity of EOs, they have been exploited by humans for different purposes for centuries. The most known are applications in the cosmetic and medicinal fields, as food preservatives, or insecticides in agriculture [2].

Despite the great interest in EOs among the scientific community, many sources have not been investigated, mainly due to the low yields associated with conventional extraction methods like distillation, cold pressing, or solvent extraction. Consistently, new innovative and more efficient techniques have emerged, such as supercritical fluid extraction and microwave-assisted extraction. This latter technique is of particular interest because it offers the possibility to perform solvent-free microwave extraction (SFME), enhancing both the efficiency and the sustainability of the process [3]. A work comparing SFME with conventional hydrodistillation highlighted that SFME provides EOs with higher quality, requires a lower amount of energy, and has shorter extraction times [4].

In the present work, we applied SFME to extract EOs from *Papaver rhoeas* L., an engaged and still underestimated plant. 

**Figure 1:**
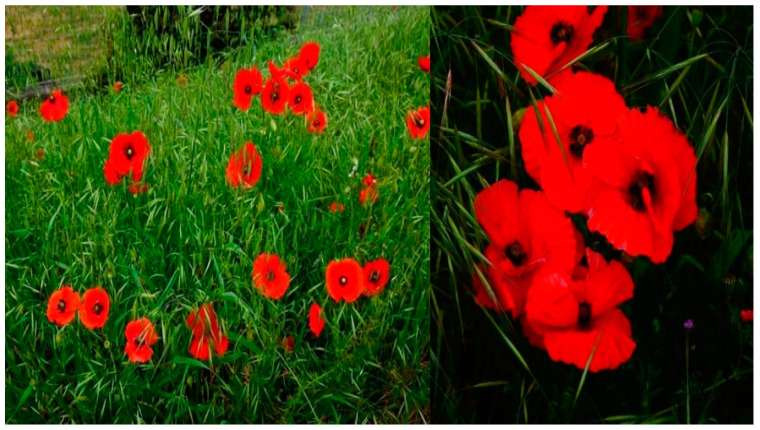
*Papaver rhoeas* in its natural habitat in Borgo Priolo, Lombardy, Italy (photograph by Dr. Luca Bernasconi).

*Papaver rhoeas* L. (Papaveraceae), also known as common poppy, corn poppy, corn rose, field poppy, Flanders poppy, red poppy, Odai, or rosolaccio, is an herbaceous annual aromatic plant native to Eurasia and North Africa. It has an erect, thin, strong, slightly hairy stem, and it is up to 60 cm tall. The plant possesses serrated, light green leaves. The flowers have scarlet-red petals with a black base [5]. The species, widespread in Italy and considered an archaeophyte, normally grows in fields and on the edges of roads and railways (Figure 1). Although it used to be considered a common cereal weed, nowadays, it is no longer easily found due to the systematic use of herbicides. 

*P. rhoeas* has a long-lasting tradition of use in folk medicine, whose applications depend on the considered country. Its most widespread use can be observed in Turkey, where the herb is exploited for its sedative activity, the fruit and seed are used against gastrointestinal diseases, and many preparations can be made for external uses [6]. In Italy, its flowers exert sedative, relaxant, antispasmodic, and emollient action, promoting sweating in case of fever, while the fruit and young shoots were traditionally exploited for their sedative, hypnotic, and depurative functions [6,7]. They are effective for treating colic, anxious states, tonsillitis, bronchitis, and nervous cough. It is also a mild sleeping agent [8,9]. In Iran, the seed and capsule are used as antidiabetics, while the flower is used for addiction treatment, sedative, and hypnotic purposes. Other applications of the aerial parts can also be found in Algeria (against respiratory diseases), Cyprus (to improve nervous/mental conditions, digestive), and Spain (against respiratory diseases) [6].

Furthermore, due to their characteristic red color, their extracts are used as a textile dye [10]. A more recent application foresaw the use of this characteristic to detect fingerprints using daylight imaging [11].

Researchers investigated the non-volatile fractions obtained from *P. rhoeas*, highlighting that the activities are mainly given to isoquinoline-based alkaloids, including rhoeadine, rhoeagenine, allotropine, berberine, coptisine, sinactine, and isocorhydine [12,13]. The red color is due to the presence of anthocyanins such as delphinidin-3-O-glucoside, cyanidin glycosides, peonidin-3-O-glucoside, petunidin glycosides, and delphinidin-3-p-coumaroylglucoside [14].

Conversely, the volatile fractions from *P. rhoeas* are still poorly investigated. In 2022, a work reported that EOs showed activity against parasites like nematodes and trichomonads and are exploited by animals for self-medication purposes [15]. Despite the ecological nature of these preliminary results, this work could pave the way for new applications of *P. rhoeas* EO, mainly applied to the fight against parasites affecting both the animal and plant kingdoms. To date, only one preliminary study regarding the analysis of *P. rhoeas* EO obtained by hydrodistillation is available, but a deeper investigation is necessary to establish its whole fingerprint [16].

In this work, we analyzed and characterized EO from the fresh petals and leaves of *P. rhoeas*, grown in Italy, obtained by SFME [17]. In particular, we considered the flowers and leaves of two Italian populations of *P. rhoeas*, one coming from a hilly area and the other from a lowland area of Lombardy. The present work aims to highlight differences between populations of *P. rhoeas* growing in different environments and to establish its whole fingerprint, to understand its ecological role, and to shed light on its possible applications.

## 2. Materials and Methods

### 2.1. Chemicals

Octyl octanoate (98%) internal standard (IS), alkane mix (C_6_–C_35_), dimethyl disulfide (DMDS), iodine, anhydrous sodium sulfate, and sodium thiosulfate were obtained by Sigma-Aldrich, Inc. (St. Louis, MO, USA). Dichloromethane was purchased from Merck (Darmstadt, Germany). Ultrapure water (LC-MS grade) was produced using the Milli Q-Milli RO system from Millipore (Burlington, MA, USA). Diethyl ether and *n*-hexane were purchased from Carlo Erba reagents (Milano, Italy). Cantharidin from *Cantharis vescicatoria* (0.5% in ethanol) was purchased from Galeno s.r.l. (Comeana, Italy)

### 2.2. Plant Materials

Fresh petals and leaves of *P. rhoeas* were collected at the full flowering stage in May 2023 in Borgo Priolo (Lombardy, Italy, 44°58′ N 9°09′ E, 144 m a.s.l.) and Pavia (Lombardy, Italy, 45°11′07″ N 9°09′18″ E, 77 m a.s.l.). Samples were immediately refrigerated at +4 °C and subsequently stored at −20 °C until extraction. Plants were identified according to Pignatti et al.’s method [5]. Voucher specimens (PRF01, PRF02, PRL01, and PRL02) were kept in the Department of Drug Sciences of the University of Pavia, Italy. The relative humidity levels of fresh petals and leaves were determined to be 70.86% ± 1.5 and 76.55% ± 1.8 by a Sartorius moisture analyzer (Sartorius AG, Goettingen, Germany). All measurements were made in triplicate. 

### 2.3. SFME Procedure

Essential oils were isolated according to previously described methods with small modifications [18,19].

In brief, after the addition of an internal standard (octyl octanoate 0.25 mg), triplicate samples (50 g) of fresh petals and leaves of *P. rhoeas* were irradiated for 12 min (2 min up to 95 °C and held at this temperature for 10 min), without the addition of any solvents or water. Solvent-free extraction was performed using a scientific single-mode microwave apparatus (Discover^®^, CEM Corporation, Matthews, NC, USA) with a maximum power of 300 Watts. The apparatus was equipped with a cooling system using compressed air. The temperature setpoint was regulated by power feedback using an optical fiber inside the sample vessel, directly connected to a control unit system. The Pyrex sample vessel was equipped with a modified Clevenger apparatus according to Chemat et al.’s method [18]. Essential oil and aromatic water (hydrosol) were simply separated by decantation. The essential oil was collected and the residual inner plant water isolated was extracted with DCM (3 × 10 mL), dried over anhydrous Na_2_SO_4_, and concentrated under reduced pressure; finally, the solvent was completely evaporated by a gentle N_2_ stream. The overall essential oil obtained was dried under anhydrous sodium sulfate and stored in the dark and sealed at −20 °C until GC/MS analyses. 

### 2.4. Fractionation and Alkylthiolation of Alkenes 

After the analysis of the total essential oil, a portion of each sample was subjected to a selective purification process to isolate the fraction containing hydrocarbons and subsequently to conduct derivatization with DMDS [20].

### 2.5. GC/MS Analysis 

The analyses were carried out using a GC Model 6890N coupled to a benchtop MS Agilent 5973 Network (Agilent, Santa Clara, CA, USA). Chromatographic separation was performed using an Elite-5MS (5% phenyl methyl polysiloxane) capillary column of 30 m × 0.32 mm i.d. and film that was 0.32 μm thick (Agilent, Santa Clara, CA, USA). The carrier gas was He and had a flow of 1 mL/min. Aliquots of 1 μL of each essential oil, after dilution (1 mg/mL) with dichloromethane, were manually injected in splitless mode. The oven temperature program included an initial isotherm of 40 °C for 5 min, followed by a temperature ramp up to 260 °C at 40 °C/min and a final isotherm at this temperature for 10 min. Injector and detector temperatures were set at 250 °C and 280 °C, respectively. Mass spectra were acquired over the 20–400 amu range at 1 scan/sec with an ionizing electron energy of 70 eV. DMDS derivatives were analyzed by GC/MS using the same chromatographic equipment with the following operative program: samples (1.0 μL) were injected in splitless mode with a column temperature program of 70 °C for 5 min, then increased to 320 °C at 7 °C/min and finally held for 10 min. The injector and detector were set at 250 and 280 °C, respectively.

### 2.6. Identification of the Volatile Compounds

The identification of the volatile compounds was performed using retention indices (RI) and mass spectra, according to Adams [21], by comparison with a NIST database mass spectral library [22] and using published data (Pubchem, http://pubchem.ncbi.nlm.nih.gov/, access date December 2022; Flavornet, http://www.flavornet.org/, access date December 2022; ChemSpider, http://chemspider.com/, access date December 2022). The relative amount of each component was expressed as the percent peak area relative to the total peak area from GC/MS analyses of the whole extracts using the following equation:

Relative contents (%) = (area under peak/total peak area) × 100%

The RI^c^ were calculated as shown in the equation:

RI = 100 × n + [100 × (tx − tn)]/(tn + 1 − tn)

where RI is the retention index of the unknown compound x, n is the number of carbon atoms of the *n*-alkane eluted before x, n + 1 is the number of carbon atoms of the *n*-alkane eluted after x, tx is the retention time of x, tn is the retention time of the *n*-alkane eluted before x, and tn + 1 is the retention time of the *n*-alkane eluted after x.

### 2.7. Statistical Analysis

Principal Component Analysis (PCA) was performed to divide the information that was provided by compounds by sample type. Visual inspection of the deriving scree-plot allowed for the identification of the most informative subset of principal components. Data were analyzed and graphs elaborated by functions implemented in R software for statistical computing and graphics version 4.2.2 (www.r-project.org access December 2022).

## 3. Results

### 3.1. Essential Oil Characterization

SFME extraction of fresh petals yielded 0.34 ± 2.4%, while leaves yielded 0.27 ± 3.1% (weight/fresh weight basis). Essential oils obtained by SFME from fresh petals and leaves of *P. rhoeas* were analyzed via GC/MS. A total of 106 compounds were identified (Table 1), listed in order of their elution together with their retention indices (RIs) on the Elite-5 MS column, and compared to the corresponding values from the literature [21]. The alkylthiolation reaction was exploited to elucidate different isomeric structures of mono-unsaturated hydrocarbons. Thus, the so-obtained α,β-bis(methylthio) alkanes were stable under GC conditions and their MS fragmentation allowed for the correct identification of the alkene isomers. The percentage of each compound compared to the total essential oil, using octyl octanoate as an internal standard, was also reported. In Table 2, percentages related to the different classes of compounds are summarized. 

#### 3.1.1. Composition of *Papaver rhoeas* Flower EO from Hill Population

Saturated hydrocarbons (41.50%) are the most represented class of the total EO, closely followed by unsaturated hydrocarbons (41.40%). The most abundant compounds belonging to the first class are nonadecane (13.10%), heneicosane (12.95%), and tricosane (9.36%), while the ones belonging to the second class are 3-nonadecene (15.54%), 9-heneicosene (10.84%), 1-heneicosene (6.37%), and 7-tricosene (3.32%). Esters (11.60%) mainly consist of ethyl linoleate (4.57%), ethyl palmitate (3.57%), methyl palmitate (1.69%), and ethyl oleate (1.17%). Organic acids (3.41%) are represented by palmitic acid (2.12%), oleic acid (0.61%), and linoleic acid (0.48%). A total of 0.92% of the essential oil is represented by the class of aldehydes, of which the main compounds are nonanal (0.45%), decanal (0.23%), and undecanal (0.19%). Oxygenated sesquiterpenes (0.61%) are represented only by farnesol (0.61%). Alcohols constitute 0.42% of the total, of which the only ones present are 4-vinyl-2-methoxy-phenol (0.33%), and benzyl alcohol (0.08%). Oxygenated monoterpenes (0.15%) are represented by cantharidin (0.37%) and β-citronellol (0.18%).

#### 3.1.2. Composition of *Papaver rhoeas* Leaf EO from Hill Population

As for the previous case, the dominant class *P. rhoeas* leaves from the hill population are saturated hydrocarbons, representing 50.02% of the total essential oil. The most present compounds are tricosane (15.58%), heneicosane (13.92%), tetracosane (9.92%), and pentacosane (4.15%). The second most abundant class of compounds is represented by esters (14.87%), including ethyl hexadecanoate (11.79%), ethyl linoleate (1.20%), ethyl linolenate (1.06%), and methyl hexadecanoate (0.68%). Unsaturated hydrocarbons (12.64%) are dominated by 9-heneicosene (3.80%), 9-tricosene (2.24%), and 10-docosene (1.15%). Oxygenated diterpenes (7.36%) are represented by phytol (5.82%) and isophytol (1.54%). The class of organic acids represents 4.47% of the essential oil, of which the main compounds are palmitic acid (4.06%) and linoleic acid (0.41%). This is followed by the class of aldehydes, which constitutes 2.71% and consists mainly of phenylacetaldehyde (1.75%), β-cyclocitral (0.51%), and tetradecanal (0.45%). Oxygenated monoterpenes (1.95%) are represented by nerol (0.49%), geraniol (0.41%), *trans*-β-damascenone (0.34%), and geranylacetone (0.29%). The less well-represented classes are oxygenated sesquiterpenes (0.68%), mainly represented by β-ionone (0.34%), dihydroactinidiolide (0.34%), and the ketones class (0.49%).

#### 3.1.3. Composition of *Papaver rhoeas* Flower EO from Lowland Population

**Figure 2:**
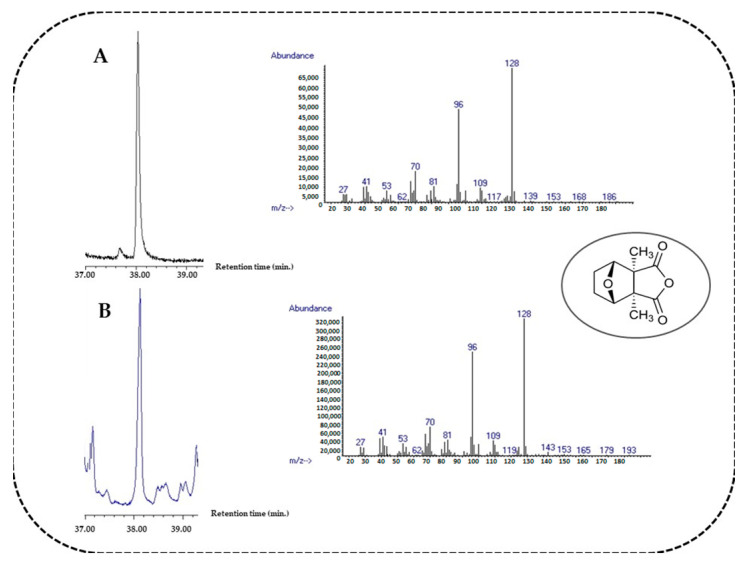
The chromatographic profile and mass spectrum of the cantharidin reference compound (panel **A**) and cantharidin in the EOs of flowers of *P. rhoeas* (panel **B**).

*P. rhoeas* flowers from the lowland population makes the EO richest in saturated hydrocarbons, representing 56.91% of the total essential oil. The most presented compounds are heneicosane (17.43%), tricosane (13.30%), nonadecane (10.05%), and tridecane (7.00%). As for the EO obtained from the flowers of hill populations, the second most abundant class of compounds is represented by unsaturated hydrocarbons (14.84%), among which 9-heneicosene (4.67%), 3-nonadecene (3.47%), and 9-tricosene (1.74%) stand out. Aldehydes constitute 10.38% of the total essential oil, and the main representative compounds are nonanal (3.10%), heptanal (2.00%), and undecanal (1.26%). Acids constitute 6.82% and mainly consist of palmitic acid (2.54%), oleic acid (0.75%), and linoleic acid (0.66%). A total of 4.75% of the essential oil is represented by the class of alcohols, where the main compounds are 2-phenylethanol (3.72%), benzyl alcohol (0.47%), and 2-nonenol (0.38%). The ester class (2.65%) is mainly represented by ethyl hexadecanoate (1.49%), ethyl linoleate (0.54%), and methyl octadecanoate (0.23%). Oxygenated monoterpenes (1.57%) are represented by β-citronellol (0.93%), geraniol (0.55%), and cantharidin (0.09%). The presence of cantharidin is confirmed using the pure reference standard, analyzed according to the previously described method. Figure 2 shows that cantharidin (Figure 2A) has the same retention time as that displayed in the essential EO samples (Figure 2B). Furthermore, the mass spectrum of the sample shows specific fragment ions of cantharidin (*m*/*z* 128, 96, 70) [23,24]. It confirms the presence of the compound in the essential oil of the flowers of both populations. Oxygenated sesquitepenes constitute 1.45% of the total, of which the most abundant are γ-eudesmol (0.75%), eudesm-7(11)-en-4-ol (0.41%), and farnesol (0.30%). The class of sesquiterpenes (non-oxygenated) constitutes 0.69% and is represented by β-caryophyllene (0.46%) and α-caryophyllene (0.23%).

#### 3.1.4. Composition of *Papaver rhoeas* Leaf EO from Lowland Population 

Unlike previous EOs, the dominant class of *P. rhoeas* leaves EO from the lowland population is that of alcohols, which represents 37.79% of the total essential oil. The compounds most present in this class are 2-phenylethanol (18.55%), benzyl alcohol (11.83%), and *cis-*3-hexenol (5.85%). The second most abundant class of compounds is represented by saturated hydrocarbons (14.64%), in which are found heneicosane (5.55%), tricosane (2.78%), and nonadecane (2.34%). The class of oxygenated diterpenes consists solely of phytol (14.14%). Unsaturated hydrocarbons (9.52%) are dominated by 1-eicosene (2.58%), 9-heneicosene (2.38%), 3-nonadecene (1.54%), and 10-docosene (1.41%). Esters characterize 8.31%, of which there are ethyl linoleate (4.23%), ethyl ester of hexadecanoate acid (2.39%), methyl ester of hexadecanoic acid (0.86%), and ethyl linolenate (0.73%). A total of 6.91% of the essential oil is represented by the aldehyde class, of which the main compounds are benzaldehyde (3.66%), phenylacetaldehyde (2.36%), and tetradecanal (0.71%). This is followed by the class of oxygenated monoterpenes that constitute 2.98% and consist mainly of geraniol (0.96%), *trans*-β-damascenone (0.60%), nerol (0.38%), and geranylacetone (0.37%). Finally, there are the classes of terpenes (1.61%) and oxygenated sesquiterpenes (1.35%) represented by neophytadiene (1.61%) and dihydroactinidiolide (0.69%) and β-ionone (0.65%), respectively.

### 3.2. Similarity Between Lowland and Hill Populations

**Figure 3:**
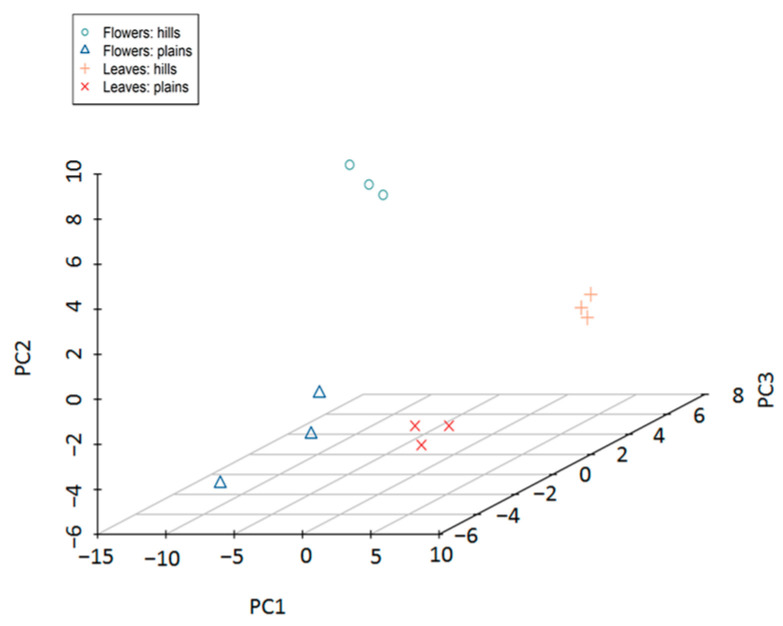
The results of PCA. Each dot corresponds to a sample, with symbols and colors differentiating among types.

Principal Component Analysis (PCA) was performed on data from 12 samples (hill flowers: *n* = 3; plains flowers: *n* = 3; hill leaves: *n* = 3; plains leaves: *n* = 3) corresponding to 111 compounds with the aim of providing a graphical representation of the similarity between samples. The three-dimensional plot in Figure 3 describes graphically the similarity between samples divided by the first three principal components (PCs) selected as the most informative based on the visual inspection of the scree plot. The first principal component (PC1) is effective in differentiating flowers from leaves, while PC2 discriminates samples from plains from those from hills.

The average value of the three samples by sample type has been estimated for each of the 111 compounds. The correlation between average measures was stronger between hill and plains leaves (Spearman correlation coefficient r = 0.65) compared to the correlation estimated between hill flowers and plains flowers (r = 0.41).

**Figure 4:**
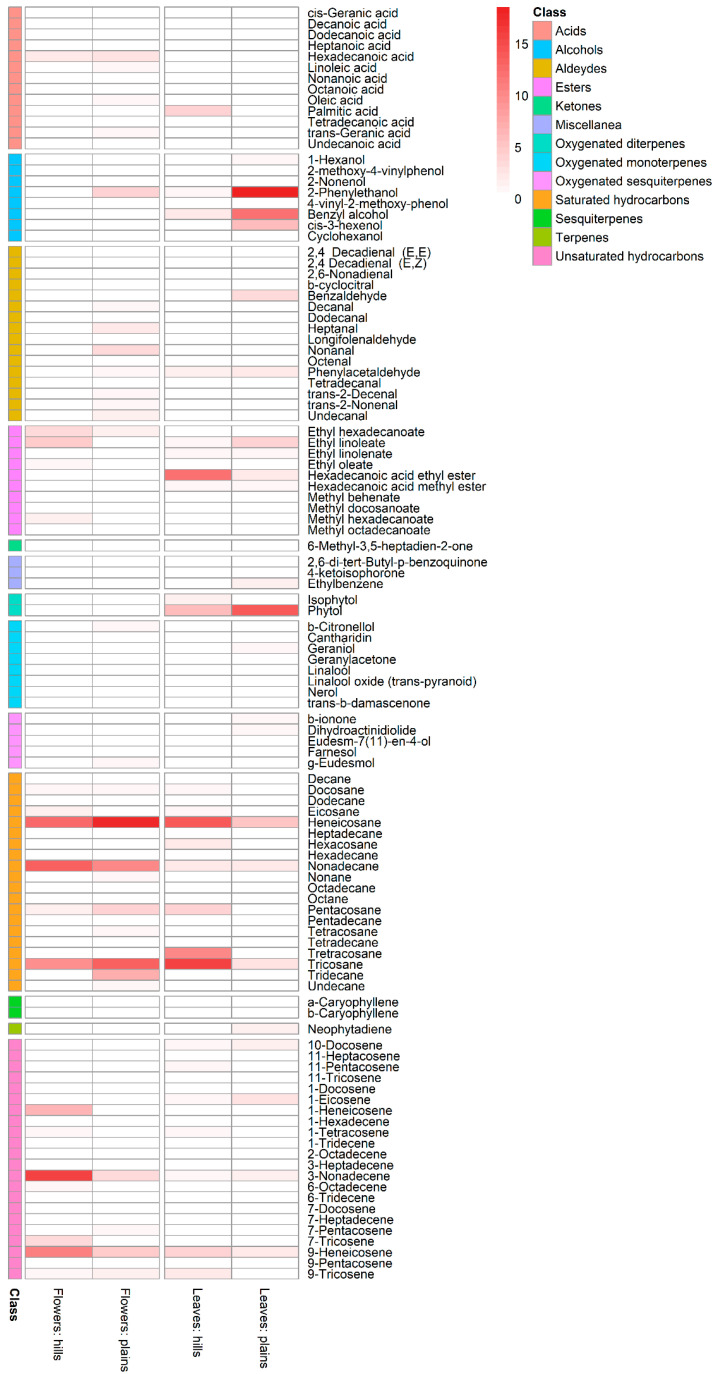
Heatmap describing compound quantity by sample type. The different shades of red in the heatmap indicate the different quantities of each compound.

The heatmap in Figure 4 provides a graphical representation of the compound quantity in the four sample types by class.

The difference in terms of mean value by compound has been estimated between hill flowers and plains flower values and between hill leaves and plains leaves.

**Figure 5:**
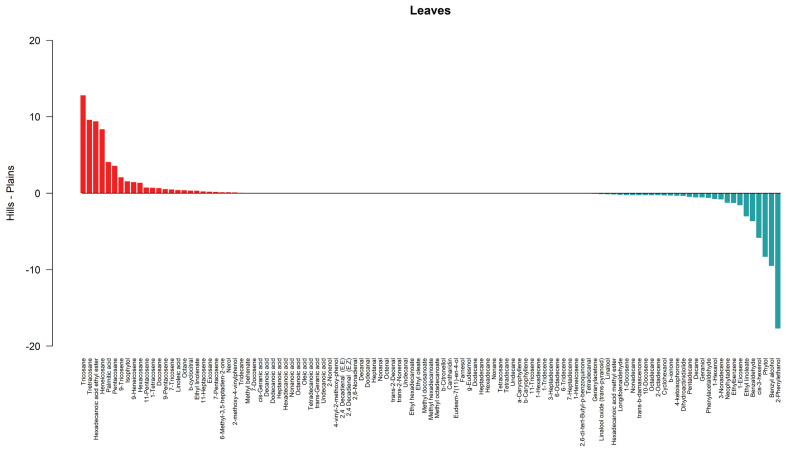
Differences in the mean value by compound between hill and plains leaf samples. Compounds characterized by higher quantities detected in hill samples compared to plains samples are depicted by red bars, and compounds characterized by lower quantities detected in hill samples compared to plains samples are depicted by blue bars. The horizontal bar in black corresponds to a condition of no difference in terms of mean value.

**Figure 6:**
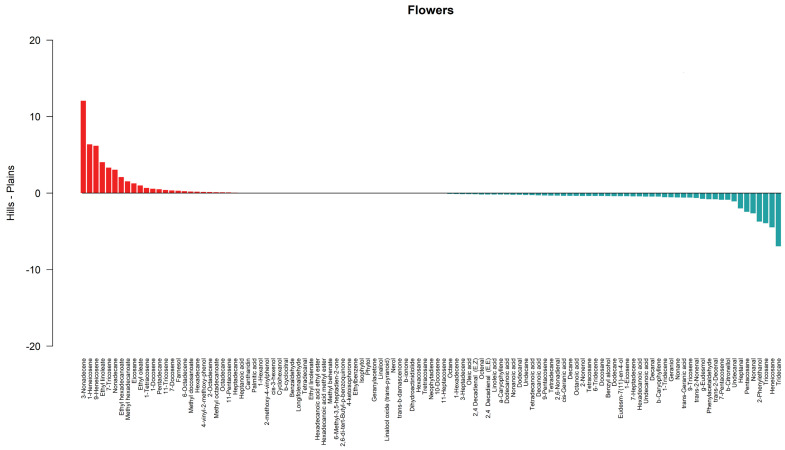
Differences in terms of mean value by compound between hill and plains flower samples. Compounds characterized by higher quantities detected in hill samples compared to plains samples are depicted by red bars, and compounds characterized by lower quantities detected in hill samples compared to plains samples are depicted by blue bars. The horizontal bar in black corresponds to a condition of no difference in terms of mean value.

The barplots in Figure 5 and Figure 6 describe graphically the differences in terms of means between sample types for leaves and flowers, respectively. Compounds characterized by higher quantities detected in hill samples compared to plains samples are depicted by red bars, and compounds characterized by lower quantities detected in hill samples compared to plains samples are depicted by blue bars.

## 4. Discussion

Essential oils extracted from *P. rhoeas* have been largely underestimated but have gained more and more attention in recent years. The reasons for this rekindled interest are mainly due to the ecological role of these volatile fractions, even if recent evidence could also pave the way for new applications of *P. rhoeas* EO as an antiparasitic agent. Consistently, we focused on the flowers and leaves of *P. rhoeas* from hilly and plain territories. 

EOs were isolated by SFME, an extraction technique based on the combination of microwave heating and solvent-free distillation. Advantages related to this innovative technique mainly concern the low time and energy required and the higher efficiency of extraction [4]. All these characteristics allow for the reduction in disadvantages often related to the nature-aided drug discovery process, which involves a prolonged time of extraction and a high amount of biomass. Due to the low yields (lower than 0.5%), in this case, EOs were separated from hydrosol via liquid/liquid extraction before GC/MS analysis. Furthermore, the so-obtained EOs were subjected to an alkylthiolation reaction to elucidate the correct isomers of alkenes present in the phytocomplex.

**Figure 7:**
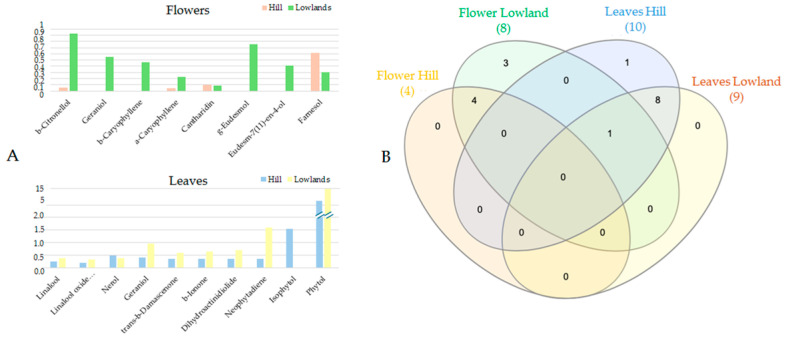
Contents of terpenes in different samples expressed as % (**A**) and Venn diagram with the number of compounds shared between different samples (**B**) of *P. rhoeas*.

Saturated hydrocarbons represent the dominant class of the volatile fraction of flowers (both hill and plain) and hill leaves (range 41.50–56.91%), and the main compounds were tricosane, heneicosane, tetracosane, pentacosane, nonadecane, and tridecane, distributed differently among the different sampled populations. These compounds are the main constituents of epicuticular waxes that protect the plant from environmental stresses such as light and heat, as well as preventing the loss of liquids by the cells. However, the activity of saturated hydrocarbons is not limited to the defense of the plant, but they are also involved in the attraction of pollinators. Differently, the leaves from the lowland population had alcohols as the dominant class (37.79%), while saturated hydrocarbons constituted only 14.64% of the EO. Conversely, unsaturated hydrocarbons are variously abundant (range 9.52–41.40%), and saturated hydrocarbons are involved in the attraction of pollinators. Thus, molecules belonging to this class can mimic the sexual pheromones emitted by female pollinating insects, triggering attraction in male insects. Since *P. rhoeas* does not have a food reward, the production of these compounds is of pivotal importance to attract pollinating insects [25,26]. Under a pharmacological point of view, of particular interest is the class of terpenoids, with this class of secondary metabolites being endowed with several biological effects [27,28]. In the present work, 17 different terpenes and terpenoids have been identified, being differently distributed among samples. A graphical representation of both quantitative (panel A) and qualitative (panel B) differences is reported in Figure 7 [29].

The presence of cantharidin in the flowers of both populations studied is of particular interest, since this oxygenated monoterpene is usually produced by insects both for defensive and attractive purposes. This metabolite is a toxic vesicant present in the blisters of species belonging to the family Malidae, with concentrations being higher in females [30,31]. It causes a strong irritation of skin and mucous membranes, but it also has some applications in folk medicine. Thus, cantharidin is used as an anticancer agent in traditional Asian medicine, even if its administration is limited by its severe side effects [32,33]. The identification of cantharidin in *P. rhoeas* flowers is of particular interest because the plant kingdom usually produces its natural analog, palasonin, and, to the best of our knowledge, this is the first time that cantharidin has been identified in a plant extract [34]. The presence of cantharidin in *P. rhoeas* flowers may be explained by considering their attractive roles, since pollinating insects can be attracted by this substance for self-protection through ingestion or because it can work as a pheromone of aggregation and sexual communication. Furthermore, there may be further unknown natural ecological interactions between apparently unrelated species that use cantharidin [35].

## 5. Conclusions

In conclusion, in the present work, we add further content to the characterization of the volatile fraction of *P. rhoeas*. Differences among EOs obtained for flowers and leaves and from hill and lowland populations have been evaluated, highlighting the influence of the habitat on the metabolomic profile of the considered species. Of particular interest is the identification of cantharidin in flowers, as this metabolite has never been identified in plants so far. The exploration of *P. rhoeas* essential oils can give indications of the ecological significance of antiparasitic defense and of any potential applications of such oils in various fields. Through innovative extraction techniques and meticulous analyses, we have shed light on the compositions of these volatile fractions, revealing their roles in plant defense, pollinator attraction, and potential interactions between species.

## Figures and Tables

**Table 1:** The percentage compositions of the essential oil from flowers and leaves of *Papaver rhoeas*.

			Flowers		Leaves		Identification ^(e)^
			Hill	Lowlands	Hill	Lowlands	
Compound ^(a)^	Ri ^(b)^	Ri ^(c)^	% ^(d)^	%	%	%	
Octane	800	801	0.14 ± 0.13	0.20 ± 0.27	0.39 ± 0.28	-	STD, RI
Ethylbenzene	859	861	-	-	-	1.29 ± 0.28	NIST, RI
*cis*-3-Hexenol	863	866	-	-	-	5.85 ± 0.29	NIST, RI
1-Hexanol	870	876	-	-	-	0.76 ± 0.24	NIST, RI
Cyclohexanol	891	894	-	-	-	0.30 ± 0.24	NIST, RI
Benzaldehyde	965	967	-	-	-	3.66 ± 0.34	NIST, RI
Nonane	900	901	0.09 ± 0 06	0.65 ± 0.60	-	-	NIST, RI
Heptanal	901	907	-	2.00 ± 0.20	-	-	NIST, RI
Decane	1000	1000	0.09 ± 0.11	0.46 ± 0.39	-	0.55 ± 0.41	STD, RI
Benzyl alcohol	1037	1041	0.08 ± 0.09	0.47 ± 0.45	2.31 ± 0.37	11.83 ± 0.82	NIST, RI
Phenylacetaldehyde	1048	1049	-	0.80 ± 0.36	1.75 ± 0.23	2.36 ± 0.33	NIST, RI
Octenal	1060	1061	-	0.18 ± 0.19	-	-	NIST, RI
2-Nonenol	1072	1072	-	0.38 ± 0.25	-	-	NIST, RI
Heptanoic acid	1091	1092	0.14 ± 0.10	0.09 ± 0.07	-	-	NIST, RI
Undecane	1100	1101	0.43 ± 0.71	0.68 ± 0.27	-	-	STD, RI
Linalool	1105	1101	-	-	0.23 ± 0.24	0.37 ± 0.28	NIST, RI
6-Methyl-3,5-heptadien-2-one	1106	1106	-	-	0.49 ± 0.33	0.37 ± 0.42	NIST, RI
Nonanal	1108	1111	0.45 ± 0.69	3.10 ± 0.21	-	-	NIST, RI
2-Phenylethanol	1117	1120	-	3.72 ± 0.64	0.85 ± 0.42	18.55 ± 0.42	NIST, RI
4-Ketoisophorone	1142	1149	-	-	0.26 ± 0.24	0.60 ± 0.39	NIST, RI
2,6-Nonadienal	1153	1157	-	0.33 ± 0.23	-	-	NIST, RI
*trans*-2-Nonenal	1164	1164	-	0.63 ± 0.36	-	-	NIST, RI
Linalool oxide (*trans*-pyranoid)	1177	1175	-	-	0.19 ± 0.10	0.31 ± 0.31	NIST, RI
Octanoic acid	1181	1180	-	0.36 ± 0.25	-	-	NIST, RI
Dodecane	1200	1200	0.15 ± 0.23	0.55 ± 0.46	-	-	STD, RI
Decanal	1209	1210	0.23 ± 0.28	0.69 ± 0.41	-	-	NIST, RI
β-Cyclocitral	1222	1225	-	-	0.51 ± 0.33	0.17 ± 0.08	NIST, RI
β-Citronellol	1228	1230	0.05 ± 0.03	0.93 ± 0.40	-	-	NIST, RI
Nerol	1232	1228	-	-	0.49 ± 0.48	0.38 ± 0.24	NIST, RI
Geraniol	1250	1255	-	0.55 ± 0.37	0.41 ± 0.38	0.96 ± 0.22	NIST, RI
*tran*s-2-Decenal	1265	1268	-	0.80 ± 0.44	-	-	NIST, RI
Nonanoic acid	1280	1284	-	0.21 ± 0.17	-	-	NIST, RI
6-Tridecene	1286	1290	-	0.38 ± 0.27	-	-	NIST, RI
1-Tridecene	1293	1294	-	0.53 ± 0.45	-	-	NIST, RI
Tridecane	1300	1300	0.05 ± 0.03	7.00 ± 0.80	0.34 ± 0.23	0.29 ± 0.19	STD, RI
Undecanal	1314	1313	0.19 ± 0.08	1.26 ± 0.38	-	-	NIST, RI
2-Methoxy-4-vinyl-phenol	1317	1315	-	-	0.61 ± 0.19	0.50 ± 0.33	NIST, RI
4-Vinyl-2-methoxy-phenol	1321	1319	0.33 ± 0.46	0.18 ± 0.24	-	-	NIST, RI
2,4 Decadienal (*E*,*Z*)	1326	1324	-	0.15 ± 0.08	-	-	NIST, RI
2,4 Decadienal (*E*,*E*)	1330	1337	-	0.18 ± 0.09	-	-	NIST, RI
*trans*-Geranic acid	1362	1368	0.07 ± 0.03	0.65 ± 0.29	-	-	NIST, RI
*cis*-Geranic acid	1374	1371	-	0.36 ± 0.27	-	-	NIST, RI
Decanoic acid	1373	1377	-	0.30 ± 0.37	-	-	NIST, RI
*trans*-β-Damascenone	1385	1384	-	-	0.34 ± 0.22	0.60 ± 0.32	NIST, RI
Tetradecane	1400	1400	0.04 ± 0.03	0.37 ± 0.28	-	-	STD, RI
Dodecanal	1411	1412	0.04 ± 0.02	0.26 ± 0.13	-	-	NIST, RI
β-Caryophyllene	1427	1430	-	0.46 ± 0.33	-	-	NIST, RI
Geranylacetone	1451	1450	-	-	0.29 ± 0.16	0.37 ± 0.23	NIST, RI
α-Caryophyllene	1460	1467	0.04 ± 0.02	0.23 ± 0.12	-	-	NIST, RI
2,6-di-tert-Butyl-p-benzoquinone	1468	1467	-	-	0.44 ± 0.22	0.48 ± 0.22	NIST, RI
Undecanoic acid	1468	1477	-	0.45 ± 0.06	-	-	NIST, RI
β-Ionone	1486	1484	-	-	0.34 ± 0.23	0.65 ± 0.49	NIST, RI
Pentadecane	1500	1500	0.49 ± 0.57	-	-	0.48 ± 0.19	STD, RI
Dihydroactinidiolide	1538	1542	-	-	0.34 ± 0.36	0.69 ± 0.42	NIST, RI
Cantharidin	1546	1546	0.10 ± 0.06	0.09 ± 0.03	-	-	STD, RI
Dodecanoic acid	1566	1568	-	0.19 ± 0.10	-	-	NIST, RI
1-Hexadecene	1592	1593	0.06 ± 0.03	0.18 ± 0.05	-	-	NIST, RI
Hexadecane	1600	1600	0.32 ± 0.47	0.13 ± 0.09	-	-	STD, RI
Tetradecanal	1611	1615	-	-	0.45 ± 0.33	0.71 ± 0.40	NIST, RI
γ-Eudesmol	1641	1644	-	0.75 ± 0.22	-	-	NIST, RI
7-Heptadecene	1673	1670	-	0.42 ± 0.42	-	-	NIST, RI
3-Heptadecene	1687	1681	0.45 ± 0.23	0.58 ± 0.34	-	-	NIST, RI
Heptadecane	1700	1700	0.45 ± 0.37	0.40 ± 0.20	-	-	STD, RI
Eudesm-7(11)-en-4-ol	1709	1712	-	0.41 ± 0.40	-	-	NIST, RI
Farnesol	1722	1718	0.61 ± 0.39	0.30 ± 0.19	-	-	NIST, RI
Tetradecanoic acid	1762	1763	-	0.26 ± 0.10	-	-	NIST, RI
6-Octadecene	1775	1778	0.76 ± 0.33	0.52 ± 0.34	-	-	NIST, RI
2-Octadecene	1798	1793	0.44 ± 0.47	0.30 ± 0.29	0.21 ± 0.20	0.48 ± 0.08	NIST, RI
Octadecane	1800	1800	0.31 ± 0.14	0.21 ± 0.17	-	0.27 ± 0.23	STD, RI
Neophytadiene	1837	1836	-	-	0.36 ± 0.21	1.61 ± 0.36	NIST, RI
3-Nonadecene	1881	1880	15.54 ± 1.32	3.47 ± 0.34	0.73 ± 0.29	1.54 ± 0.12	NIST, RI
Nonadecane	1900	1900	13.10 ± 0.66	10.05 ± 0.20	2.09 ± 0.40	2.34 ± 0.32	STD, RI
Methyl hexadecanoate	1933	1934	1.69 ± 0.29	0.16 ± 0.11	0.68 ± 0.19	0.86 ± 0.39	NIST, RI
Isophytol	1947	1947	-	-	1.54 ± 0.34	-	NIST, RI
Palmitic acid	1975	1974	2.12 ± 0.37	2.54 ± 0.36	4.06 ± 0.72	-	NIST, RI
1-Eicosene	1992	1994	-	0.41 ± 0.17	1.00 ± 0.38	2.58 ± 0.24	NIST, RI
Ethyl hexadecanoate	1997	1997	3.57 ± 0.49	1.49 ± 0.26	11.79 ± 0.68	2.39 ± 0.32	NIST, RI
Eicosane	2000	2000	1.26 ± 0.30	-	0.75 ± 0.40	0.55 ± 0.19	STD, RI
9-Heneicosene	2073	2074	10.84 ± 0.81	4.67 ± 0.22	3.80 ± 0.22	2.38 ± 0.23	NIST, RI
1-Heneicosene	2087	2088	6.37 ± 0.47	-	0.36 ± 0.25	0.40 ± 0.53	NIST, RI
Heneicosane	2100	2100	12.95 ± 0.80	17.43 ± 0.74	13.92 ± 0.49	5.55 ± 0.59	STD, RI
Phytol	2113	2116	-	-	5.82 ± 0.68	14.14 ± 0.41	NIST, RI
Linoleic acid	2130	2133	0.48 ± 0.38	0.66 ± 0.39	0.41 ± 0.34	-	NIST, RI
Methyl octadecanoate	2133	2136	0.34 ± 0.39	0.23 ± 0.17	-	-	NIST, RI
Ethyl linolenate	2145	2140	-	-	1.06 ± 0.46	0.73 ± 0.45	NIST, RI
Oleic acid	2152	2152	0.61 ± 0.22	0.75 ± 0.52	-	-	NIST, RI
10-Docosene	2160	2162	-	-	1.15 ± 0.60	1.41 ± 0.43	NIST, RI
Ethyl linoleate	2171	2173	4.57 ± 0.67	0.54 ± 0.36	1.20 ± 0.35	4.23 ± 0.20	NIST, RI
Ethyl oleate	2175	2178	1.17 ± 0.89	0.19 ± 0.17	-	-	NIST, RI
7-Docosene	2179	2181	0.34 ± 0.23	-	0.33 ± 0.42	0.31 ± 0.07	NIST, RI
1-Docosene	2192	2188	0.56 ± 0.51	-	-	0.25 ± 0.15	NIST, RI
Docosane	2200	2200	0.67 ± 0.52	1.05 ± 0.62	1.00 ± 0.38	0.36 ± 0.17	STD, RI
11-Tricosene	2261	2258	0.39 ± 0.52	-	-	-	NIST, RI
9-Tricosene	2279	2278	1.15 ± 0.11	1.74 ± 0.46	2.24 ± 0.14	0.18 ± 0.14	NIST, RI
7-Tricosene	2286	2281	3.32 ± 0.31	-	0.48 ± 0.21	-	NIST, RI
Tricosane	2300	2300	9.36 ± 0.64	13.30 ± 0.56	15.58 ± 0.46	2.78 ± 0.26	STD, RI
1-Tetracosene	2390	2382	0.66 ± 0.54	-	0.70 ± 0.45	-	NIST, RI
Tetracosane	2400	2400	0.29 ± 0.17	0.67 ± 0.49	9,92 ± 0.30	0.34 ± 0.24	STD, RI
11-Pentacosene	2469	2466	0.35 ± 0.47	0.25 ± 0.30	0.72 ± 0.35	-	NIST, RI
9-Pentacosene	2474	2472	0.20 ± 0.20	0.52 ± 0.52	0.52 ± 0.16	-	NIST, RI
7-Pentacosene	2480	2495	-	0.87 ± 0.61	0.16 ± 0.12	-	NIST, RI
Pentacosane	2500	2500	1.30 ± 0.45	3.76 ± 0.83	4.15 ± 0.16	0.59 ± 0.75	STD, RI
Methyl docosanoate	2527	2530	0.26 ± 0.16	0.05 ± 0.04	0.13 ± 0.09	0.10 ± 0.08	NIST, RI
11-Heptacosene	2670	2671	-	-	0.24 ± 0.21	-	NIST, RI
Heptacosane	2700	2700	-	-	1.88 ± 0.81	0.54 ± 0.73	STD, RI

^(a)^ Compounds are listed in order of elution from an Elite-5 column. ^(b)^ Retention indices according to Adams [21], unless stated otherwise. ^(c)^ Retention index determined on an Elite-5 column using a homologous series of *n*-hydrocarbons. ^(d)^ Mean ± sd of three replicates. ^(e)^ Method of identification: STD, pure compound; MS, mass spectrum; NIST, comparison with library; RI, retention indices in agreement with the literature values.

**Table 2:** The percentages related to the different classes of compounds in the essential oils from the flowers and leaves of *Papaver rhoeas*.

		Flowers %		Leaves %	
		Hill	Lowlands	Hill	Lowlands
Acids		3.41	6.82	4.47	-
Alcohols		0.42	4.75	3.76	37.79
Aldehydes		0.92	10.38	2.71	6.91
Esters		11.60	2.65	14.87	8.31
Ketones		-	-	0.49	0.37
Saturated hydrocarbons		41.50	56.91	50.02	14.64
Unsaturated hydrocarbons		41.40	14.84	12.64	9.52
Terpenes		-	-	0.36	1.61
Oxygenated monoterpenes		0.15	1.57	1.95	2.98
Sesquiterpenes		0.004	0.69	-	-
Oxygenated sesquiterpenes		0.61	1.45	0.68	1.35
Miscellanea		-	-	0.70	2.38
Oxygenated diterpenes		-	-	7.36	14.14

## Data Availability

Data are contained within this article.
